# Evolution of Cortical and White Matter Lesion Load in Early-Stage Multiple Sclerosis: Correlation With Neuroaxonal Damage and Clinical Changes

**DOI:** 10.3389/fneur.2020.00973

**Published:** 2020-09-04

**Authors:** Ramona-Alexandra Todea, Po-Jui Lu, Mario Joao Fartaria, Guillaume Bonnier, Renaud Du Pasquier, Gunnar Krueger, Meritxell Bach Cuadra, Marios Nikos Psychogios, Ludwig Kappos, Jens Kuhle, Cristina Granziera

**Affiliations:** ^1^Translational Imaging in Neurology (ThINk) Basel, Department of Biomedical Engineering, Basel University Hospital, University of Basel, Basel, Switzerland; ^2^Section of Neuroradiology, Department of Radiology, University Hospital of Basel, Basel, Switzerland; ^3^Neurologic Clinic and Policlinic, Departments of Medicine, Clinical Research, and Biomedicine, University Hospital Basel, University of Basel, Basel, Switzerland; ^4^Advanced Clinical Imaging Technology, Siemens Healthcare AG, Lausanne, Switzerland; ^5^Department of Radiology, University Hospital and University of Lausanne, Lausanne, Switzerland; ^6^Signal Processing Laboratory (LTS 5), École Polytechnique Fédérale de Lausanne (EPFL), Lausanne, Switzerland; ^7^Service of Neurology, Department of Clinical Neurosciences, Lausanne University Hospital and University of Lausanne, Lausanne, Switzerland; ^8^Siemens Healthcare AG, Zurich, Switzerland; ^9^Medical Image Analysis Laboratory (MIAL), Centre d'Imagerie BioMédicale (CIBM), Lausanne, Switzerland

**Keywords:** early relapsing remitting multiple sclerosis, MRI, MP2RAGE, cortical lesions, serum neurofilamants

## Abstract

**Introduction:** Changes in cortical and white matter lesion (CL, WML) load are pivotal metrics to diagnose and monitor multiple sclerosis patients. Yet, the relationship between (i) changes in CL/WML load and disease progression and between (ii) changes in CL/WML load and neurodegeneration at early MS stages is not yet established. In this work, we have assessed the hypothesis that the combined CL and WML load as well as their 2-years evolution are surrogate markers of neurodegeneration and clinical progression at early MS stages. To achieve this goal, we have studied a group of RRMS patients and have investigated the impact of both CL and WML load on neuroaxonal damage as measured by serum neurofilament light chain (sNfL). Next, we have explored whether changes in CL/WML load over 2 years in the same cohort of early-MS are related to motor and cognitive changes.

**Methods:** Thirty-two RRMS patients (<5 years disease duration) underwent: (i) 3T MRI for CL/WML detection and clinical assessment at baseline and 2-years follow-up; and (ii) baseline blood test for sNfL. The correlation between the number and volume of CL/WML and sNfL was assessed by using the Spearman's rank correlation coefficient and a generalized linear model (GLM). A GLM was also used to assess the relationship between (i) the number/volume of new, enlarged, resolved, shrunken, stable lesions and (ii) the difference in clinical scores between two time-points.

**Results:** At baseline, sNfL levels correlated with both total CL count/volume (ρ = 0.6/0.7, Corr-*P* <0.017/Corr-*P* < 0.001) and with total WML count/volume (ρ = 0.6/0.6, Corr-*P* < 0.01 for both). Baseline sNfL levels also correlated with new WML count/volume (ρ = 0.6/0.5, Corr-P < 0.01/Corr-P < 0.05) but not with new CL. Longitudinal changes in CL and WML count and volume were significantly associated with (i) sustained attention, auditory information, processing speed and flexibility (*p* < 0.01), (ii) verbal memory (*p* < 0.01); (iii) verbal fluency (*p* < 0.05); and (iv) hand-motor function (*p* < 0.05).

**Discussion**: Changes in cortical and white matter focal damage in early MS patients correlate with global neuroaxonal damage and is associated to cognitive performances.

## Introduction

Multiple sclerosis (MS) is a chronic inflammatory demyelinating disease of the central nervous system, which leads to the formation of focal demyelinating plaques in white and gray matter (1, 2). These lesions appear on a background of an inflammatory reaction—characterized by accumulation of lymphocytes and activated microglia—and show demyelination, in which axons are at least partially preserved (3). At all MS stages, white matter lesions (WML) are characterized by different levels of inflammatory activity, remyelination and axonal loss, with more evident ongoing activity in lesions of patients at early MS stages (4–7).

Cortical demyelination—which may be focal or diffuse—is also frequent in MS and present at early MS stages (8). Cortical lesions appear inflammatory and strongly associated with meningeal inflammation (8) and encompass plaques affecting both the cortex and the underlying white matter (leukocortical lesions), small perivascular lesions that completely located within cerebral cortex (intracortical lesions) and subpial cortical lesions (9, 10).

The presence and changes in cortical and white matter lesions (CL, WML) load are pivotal metrics for the management of multiple sclerosis (MS) patients (11).

The number of WML and CL in patients with suspicious symptoms of MS is a fundamental criterion for the diagnosis of the disease (12). WML number at baseline is predictive of conversion to MS at 20-years follow-up in patients with clinical isolated syndrome (13), and WML volume appears to be associated with disability, motor and cognitive outcome at long-term follow-up (14). The number of CL appears to correlate with disability and cognition in early MS stages and shows even stronger associations with those outcome measures than WML load (15). Besides, CL load is strongly and positively associated with cognitive dysfunction and with severe gray matter atrophy (10). Also, cortical pathology—better than WML load—is related to disability progression in all MS disease phenotypes (16) and extensive cortical damage at onset is associated with both florid inflammatory clinical activity and rapid occurrence of the progressive phase (16).

Regarding patient monitoring, the accumulation of focal damage (i.e., the increase in WML number) is one of the criteria that is currently used to follow-up therapy response and eventually therapy-switch in MS patients (17, 18).

Irreversible central nervous system damage occurs in the early phase of MS and significantly contributes to disability progression in later stages of the disease (19, 20). That is why it is currently accepted that early treatment favorably impacts the long-term outcomes of MS patients (17, 21, 22), reduces disability progression in patients with RRMS, and decreases the risk of developing clinically defined MS in patients with clinically isolated syndrome (23–26). Nonetheless, with the current plethora of MS therapies, it is of outmost importance to stratify patients that might benefit from more aggressive therapeutic regimens than others at early disease stages.

To date, it remains unclear (i) whether changes in CL/WML load during the first years of MS disease parallel changes in clinical outcome and (ii) whether CL/WML load in early MS is proportional to ongoing neurodegeneration.

In this work, we have assessed the hypothesis that—in early MS—the combined CL and WML load as well as the 2-years evolution of CL/WML number and volume are surrogate markers (i) of neurodegeneration and (ii) of clinical progression. To achieve this goal, we have studied a group of RRMS patients and have investigated the impact of both CL and WML load on neuroaxonal damage as measured by serum neurofilament light chain (sNfL) (27, 28). Next, we have explored whether changes in CL/WML load over 2 years in the same cohort of early-MS are related to motor and cognitive changes.

## Methods

### Population and Clinical Assessment

We performed a retrospective analysis in a cohort of patients enrolled at Lausanne University Hospital. Thirty-two early RRMS patients with <5 years disease duration were enrolled in the study (TP1) and followed up 2 years later (TP2). Inclusion criteria for patients were the following: definite MS diagnosis according to the revised McDonald criteria 2017, <5 years disease duration at enrolment, age between 20 and 70 years old and no other neurological or psychiatric disorder more than 3 months after the last relapse and/or end of corticosteroid therapy. Exclusion criteria were: claustrophobia and contraindications to MRI.

Also, at both TP1 and TP2, each of the 32 subjects underwent advanced MRI and a clinical examination, and 25 of them had blood sampled to measure sNfL levels at TP1.

Clinical assessment was performed using: (i) Expanded Disability status scale (EDSS) (29), (ii) Multiple Sclerosis Functional Composite score (MSFC) (30), (iii) Brief Repeatable Battery of Neuropsychological Tests; (BRBN) (31), (iv) Hospital Anxiety and Depression scale (HAD) (32), (v) Fatigue Scale for Motor and Cognitive functions (33). Physical disability of the patients was scored using the Expanded Disability Status Scale (EDSS). The difference between clinical scores at TP2 and TP1 (TP2–TP1) was used as a measure of clinical changes over time.

The institutional ethics review board approved the study and all patients gave their written informed consent.

### MR Imaging Acquisition

Images were acquired on a 3T scanner (MAGNETOM Trio a Tim system, Siemens Healthcare, Erlangen, Germany) using a 32-channel head coil. The imaging protocol included: Magnetization-Prepared 2 Rapid Acquisitions Gradient Echo (MP2RAGE, TR/TI1/TI2 = 5,000/700/2,500 ms, vs = 1.0 × 1.0 × 1.2 mm^3^, acquisition time: ~8 min) (34) and 3D Fluid-attenuated inversion recovery (FLAIR, TR/TE/TI = 5,000/394/1,800 ms, vs = 1.0 × 1.0 × 1.2mm^3^, acquisition time: ~6 min).

### Image Analysis

WML/CL were segmented by consensus by a neurologist and a neuroradiologist on 3D FLAIR and MP2RAGE images using ITK-SNAP [http://www.itksnap.org, (35)]. WML/CL number and volumes were then extracted from the segmented lesion masks using MATLAB.

The detection of CL and the definition of CL types was performed on MP2RAGE images, which are known to be more sensitive to cortical focal pathology than both MPRAGE and 3D FLAIR (36). Cortical lesions were segmented if they were characterized by a local cortical hypointensity on MP2RAGE compared to the surrounding gray matter and they had at least 1 mm in plane resolution and more than three pixels in size.

The experts who manually performed lesion detection were unaware of the patients clinical status and cognitive tests results.

**Figure 1:**
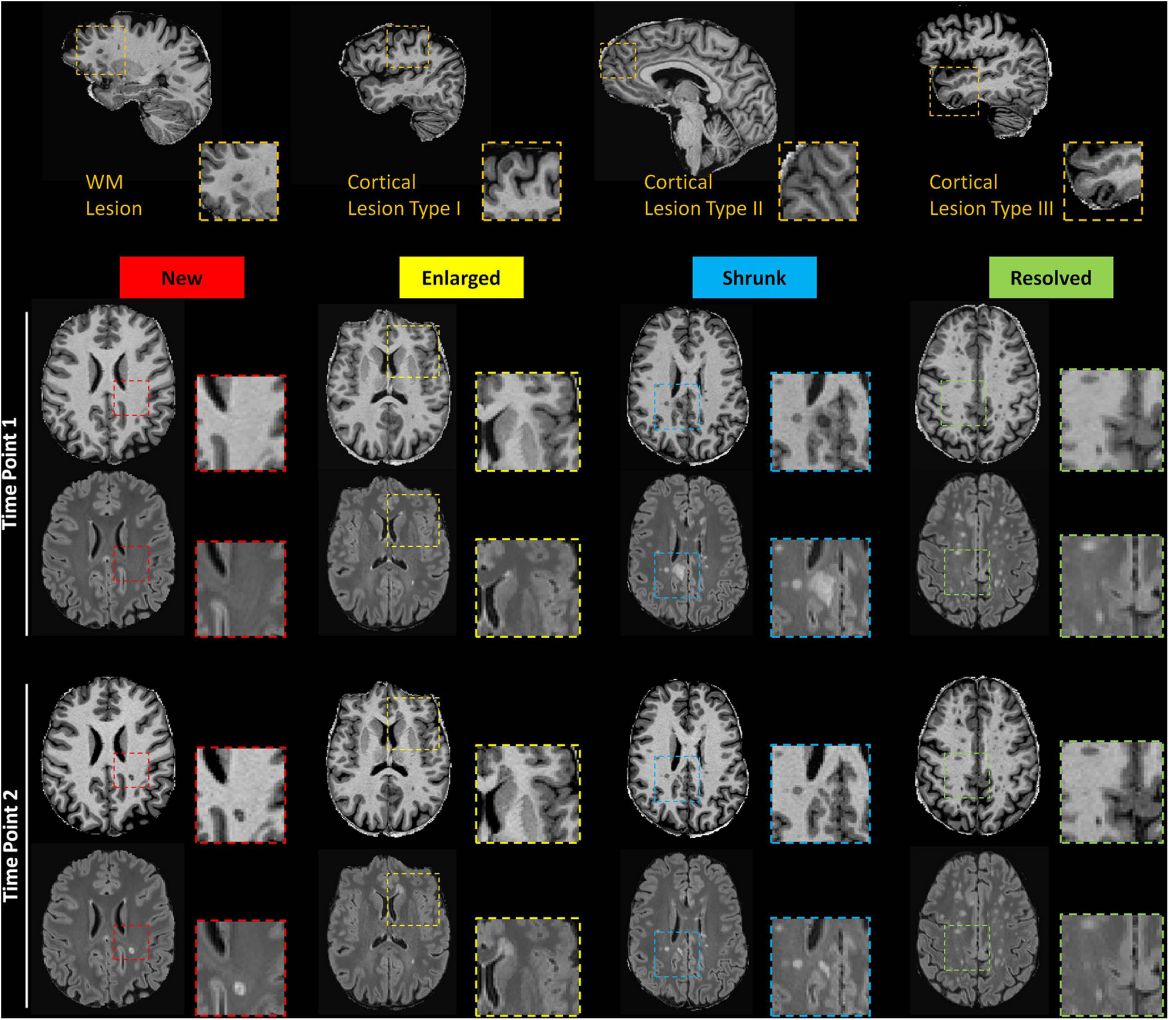
Top row: Exemplary sagittal view in one patient showing WML and CL type 1, 2, and 3. Bottom rows: Axial slices of MP2RAGE and 3D FLAIR images showing exemplary new, enlarged, shrunken and resolved WML as automatically detected.

MS lesions were then classified in five groups as proposed in (37) depending on their evolution between the two time-points: *new* (identifiable on the TP2 images but not on the TP1 images); *enlarged* (characterized by a diameter increased at TP2 by at least 50%); *resolved* (clearly visible on the TP1 images but not on the TP2 images); *shrunken* (characterized by a diameter decrease at TP2 by at least 50%); *stable*: do not follow any of the above criteria (Figure 1). For the segmentation of new, resolved, shrunken, enlarged and stable lesions, we applied an automated method developed in house (38).

### Serum Neurofilaments Measuring

Serum neurofilament light chain levels were measured using an electrochemiluminescence-based immunoassay (27).

### Statistical Analysis

Assessment of the relationship between (i) CL/WML load at baseline and baseline sNfL and (ii) 2-years changes in CL/WML load and baseline sNfL

In patients, we performed Spearman's correlations between baseline sNfL and baseline number/volume of CL/WML. We also performed Spearman's correlations between baseline sNfL and changes in number/volume of CL/WML at 2-years follow-up. *P*-values were obtained from the permutation test with a case resampling rate of 10,000. False discovery rate correction was performed by using the Benjamin-Hochberg procedure to account for multiple comparisons.

A univariate general linear model (GLM) was also performed to assess the relative contribution of CL and WML to sNfL variations, which were transformed by Box-Cox transformation to be normally distributed since the *p*-value of the Shapiro-Wilk test on the sNfL is <0.001. The best GLM model was selected by Akaike information criterion (AIC) to reduce the risks of overfitting and underfitting.

Assessment of the relationship between changes in CL/WML load and clinical changes

General linear model was performed using: (i) the number of new, enlarged, resolved, shrunken, stable lesions as well as the volume of new, enlarged, resolved, shrunken, stable lesions as predictors and (ii) the delta (TP2-TP1) of each cognitive, motor and disability score as outcome. We checked the delta of all measures for normality by the Shapiro-Wilk test and the following were Box-Cox transformed: PASAT, SRT-LTS, SRT-D, and SDMT. The delta of each measure to be transformed was rendered positive by subtracting the minimum of the delta and adding 0.01^*^ the maximum of the delta to avoid having negative values in the Box-Cox transformation. Age, gender, number of education years, and the change of the anxiety and depression scores were considered as covariates. This cohort of stable patients did not exhibit any relapses between TP1 and TP2. Backward-stepwise analyses based on AIC were performed to select the best prediction model for each clinical score. Bonferroni correction was applied to correct for the familywise error rate. A leave-one-out cross-validation (LOOCV) was conducted to assess the prediction quality of each model measured by the Spearman's correlation coefficient between the true and predicted outcomes in the validation sets of all folds.

Statistical analysis was performed using the R-project for statistical computing (https://www.r-project.org/).

## Results

Our cohort of RRMS patients consisted of 32 subjects, 13 males, 19 females with age at enrollment 35 ± 9.9 years (mean ± standard deviation, range 20–70 years); follow-up interval 21.4 ± 2.5 months, (mean ± standard deviation, range 16–27 months). All patients were <5 years from initial symptoms 32 ± 21.6 months (mean ± standard deviation, range 3–70 months) and disease diagnosis 26 ± 19.3 months (mean ± standard deviation, range 0–59 months) at TP1. 88% of patients were on treatment at the baseline and 94% on treatment at the follow-up.

At baseline, 76% of patients (*n* = 24) were on Interferon Beta, 15% (*n* = 5) on Fingolimod and 9% (*n* = 3) on Glatiramer acetate. Treated patients remained on the same treatment for the entire duration of the study. There was no corticosteroid therapy within the 3 months preceding the enrollment and follow-up MRI.

Clinical scores at the time of enrollment (TP1), at the follow-up (TP2) and the difference in clinical scores between the two time-points (TP2-TP1) as a measure of clinical changes over time are shown in Table 1.

**Table 1:** Clinical scores at the time of enrollment (TP1), at the follow-up (TP2) and the difference in clinical scores between the two time-points (TP2-TP1) as a measure of clinical changes over time.

		**TP1**	**TP2**	**TP2-TP1**
Disability and motor function	EDSS	1.6 ± 0.3	1.7 ± 0.5	—
	9-HPT (Arm function)	19.8 ± 2.8	19.6 ± 2.7	−0.21 ± 2.13
	T25FWT (Leg function)	4 ± 0.8	3.4 ± 0.5	−0.62 ± 0.69
Cognition (BRB-N)	PASAT (cognitive)	46.8 ± 10.4	48.7 ± 11.1	1.84 ± 6.71
	SRT-LTS (verbal memory)	62.3 ± 7.2	65.7 ± 5	3.41 ± 6.32
	SRT-CLTR (verbal memory)	57.6 ± 11.4	61.6 ± 10	3.94 ± 9.15
	SRT-D (verbal memory)	11.4 ± 1.1	11.8 ± 0.5	0.38 ± 1.1
	SDMT (attention)	60.5 ± 17.3	57.2 ± 11.6	−3.34 ± 18.7
	SPART10/36 (visuospatial memory)	23.2 ± 4.3	23.2 ± 3.9	−0.03 ± 4.07
	WLG (verbal fluency)	27.6 ± 5.4	27.4 ± 7.9	−0.19 ± 5.4
Mood and fatigue	HAD-A (anxiety)	6 ± 4.1	5.7 ± 3.8	−0.28 ± 3.34
	HAD- D (depression)	2.9 ± 2.4	2.1 ±2.1	−0.78 ± 2.56
	FMSC-Cognitive	23 ± 8.4	22.7 ± 9.6	−0.31 ± 7.33
	FMSC-Motor	22.7 ± 9.6	23.1 ± 10.9	−0.84 ±7.41

*Values are expressed in mean ± standard deviation unless otherwise indicated*.

*BRB-N, Brief repeatable battery of neuropsychological tests; EDSS, Expanded Disability Status Scale; T25FW, Timed 25-Foot Walk—leg function; 9-HPT, 9 Hole Peg Tests -arm function; BRB-N, Brief Repeatable Battery of Neuropsychological Tests; PASAT, paced auditory serial addition test; SRT-LTS, selective reminding test-long term storage; SRT-CLTR, selective reminding test—Consistent long-term storage; SRT-D, selective reminding test-delayed; SDMT, symbol digit modalities test; cSPART 10/36, Spatial Recall Test; WLG: word list generation; HAD-A, Hospital Anxiety and Depression scale- Anxiety; HAD-D, Hospital Anxiety and Depression scale-Depression; FMSC, Fatigue scale for Motor and Cognitive functions*.

### Longitudinal Changes in CL/WML

**Figure 2:**
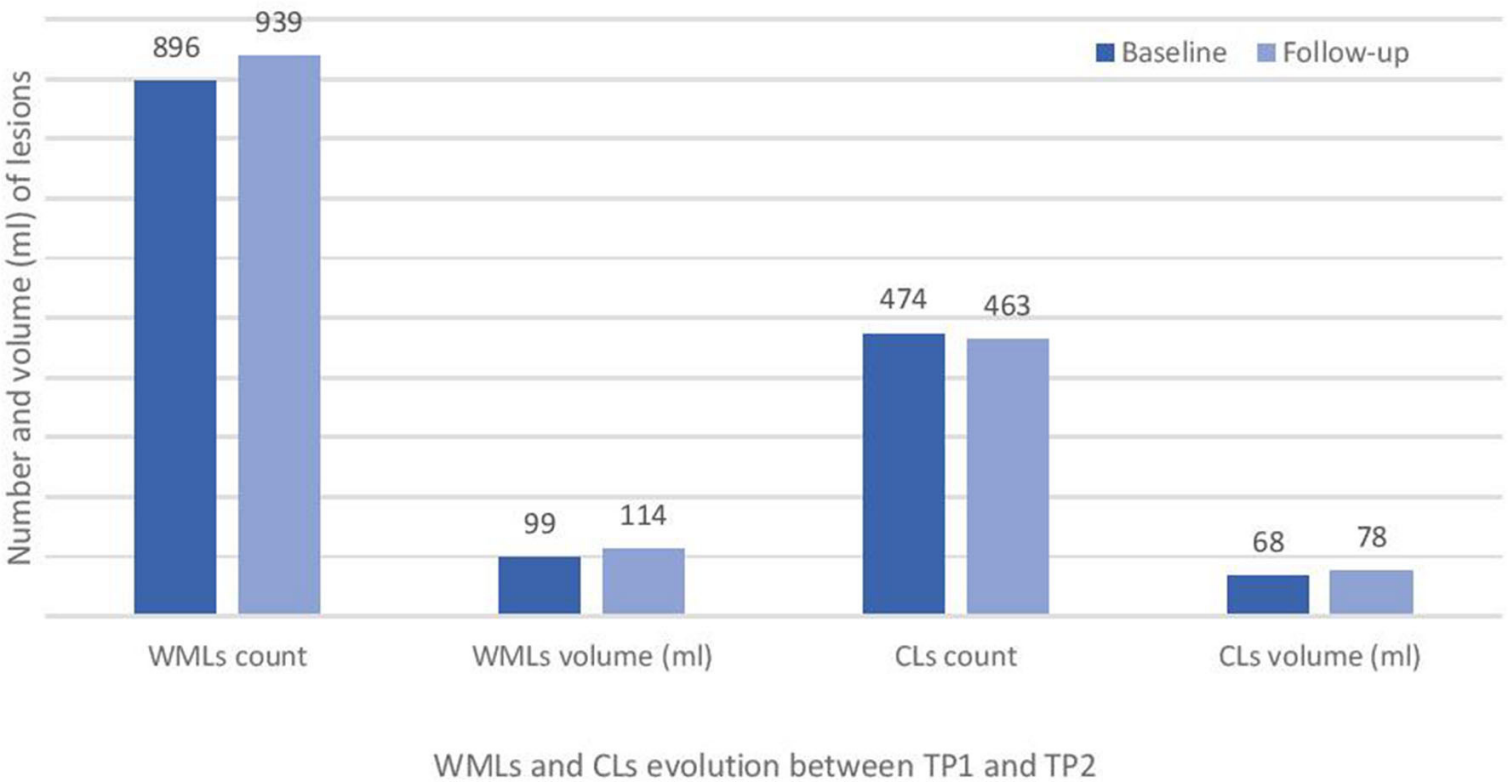
Total white matter lesions (WMLs) and cortical lesions (CLs) number at baseline (TP1) and at 2 years follow-up (TP2) in the studied cohort of RRMS patients.

**Figure 3:**
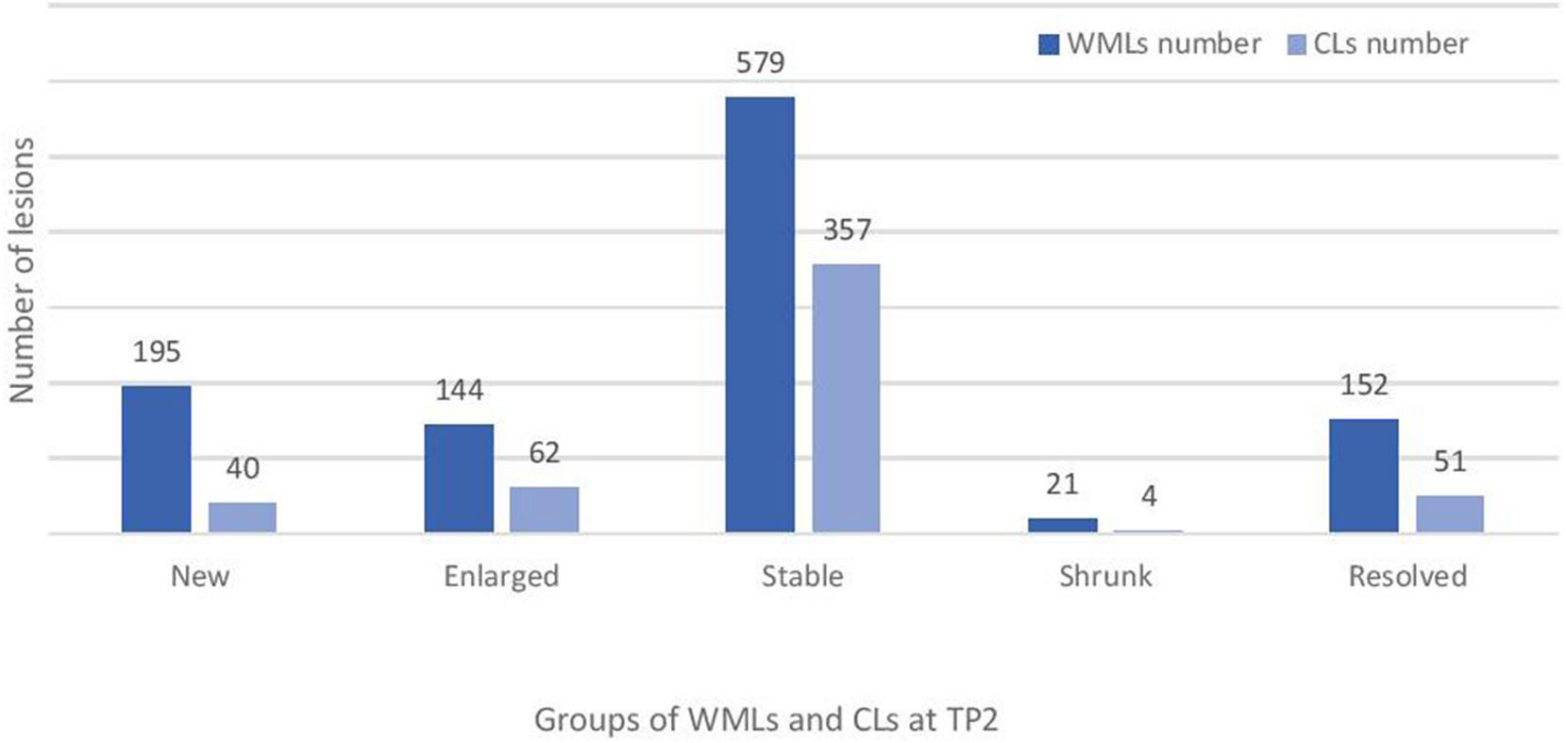
Total number of new, enlarged, stable and resolved cortical and white matter lesions at follow-up (TP2) in the studied cohort of RRMS patients. WMLs, white matter lesions; CLs, cortical lesions.

Baseline numbers and changes in WML and CL number over 2 years are reported in Figures 2, 3.

### Correlation Between sNfL and CL/WML at Baseline and With Changes in CL/WML Over 2 Years

At baseline, 164 (80.4%) of CL were type 1, 39 (19.1%) were type 2 and 1 only of type 3 (0.5%) in patients having measured sNfL. The sNfL levels in MS patients correlated with total CL count/volume (ρ = 0.6/0.7, Corr-P <0.01/Corr-P <0.001) to a similar extent than with total WML count/volume (ρ = 0.6/0.6, Corr-P <0.01 for both), Table 2. Specifically, sNfL correlated with both CL-type I number/volume (ρ = 0.5/0.6, Corr-P <0.05/Corr-P <0.01) and with CL- type II number/volume (ρ = 0.5/0.5, Corr-P <0.05 for both), Table 2.

**Table 2:** Correlation between baseline sNfL with baseline WML/CL and new WML/CL load.

**Number**			**Volume**		
	**Spearman ρ**	***P*-value**	**Corr-P**	**Spearman ρ**	***P*-value**	**Corr-P**
WML	0.58	0.003	**0.003**	0.61	0.002	**0.003**
CL	0.60	0.002	**0.003**	0.69	0.0002	**0.0006**
Lesion Type 1	0.54	0.005	**0.01**	0.64	0.0008	**0.003**
Lesion Type 2	0.45	0.025	**0.025**	0.50	0.013	**0.018**
New WML	0.58	0.002	**0.009**	0.51	0.01	**0.022**
New CL	0.38	0.06	0.085	0.31	0.13	0.13

*P-values and Corr-P given are before and after Benjamini-Hochberg procedure, respectively. Bold values indicates the statistically significant values*.

The best GLM model included CL count/volume and WML volume as predictors and revealed a moderate association between sNfL at baseline and WML/CL volume (adj-*R*^2^ = 0.5, *p* = 0.0006, pred-*R*^2^ = 0.09). Besides, sNfL levels at baseline correlated with new WML count/volume (ρ = 0.6/*p* = 0.5, *p* = 0.002/*p* = 0.01, Corr-P <0.01/Corr-P <0.05) but not with new CL count/volume, Table 2.

### Correlation Between Changes in CL/WML and Changes in Clinical Scores

Table 3 shows that the longitudinal changes in CL and WML volume and number were significantly associated with changes in:

- Hand function (9HPT, adj-*R*^2^: 0.5, Corr-*P* = 0.03, and ρ = 0.5 after leave-one-out cross-validation, LOOCV)
- Sustained attention, auditory information, processing speed and flexibility (PASAT, adj-*R*^2^: 0.5, Corr-*P* = 0.01, and ρ = 0.5 after leave-one-out cross-validation, LOOCV)
- Verbal memory (SRT-D, adj-*R*^2^: 0.5, *P* = 0.01 and ρ = 0.45 after LOOCV)
- Semantic verbal fluency (WLG- word list generation test, adj-*R*^2^: 0.5, Corr-*P* = 0.05, and ρ = 0.4 after LOOCV)

**Table 3:** Multiple regression between change of MRI metrics and change of clinical scores.

	**Stepwise regression**				**LOOCV**		
	**Adjusted-*R*^**2**^**	***P*-value**	**Minimum, maximum, lambda**	**Corr-P**	**Spearman ρ**	***P*-value**	**Corr-P**
T25FWT	0.07	0.233	—	1	—	—	—
**9-HPT**	0.48	0.003	—	**0.03**	0.52	0.002	**0.02**
**PASAT**	0.46	0.001	(−11,24,0.4)	**0.01**	0.56	0.001	**0.008**
SRT-LTS	0.39	0.004	(−8,21,0.4)	**0.04**	0.4	0.025	0.2
SRT-CLTR	0.24	0.03	—	0.27	0.43	0.013	0.1
SRT-D	0.57	0.0003	(−2,4,0.8)	**0.003**	0.45	0.009	0.08
SPART 10/36	0.07	0.074	—	0.66	—	—	—
**WLG**	0.43	0.003	—	**0.03**	0.64	0.0001	**0.001**
SDMT	0.04	0.146	(−96,16,2.4)	1	—	—	—

*Stepwise regression: The given P-values and Corrected P-values (Corr-P) are before and after Bonferroni correction, respectively. LOOCV: Spearman's rank correlation coefficient (ρ) between real and predicted outcome obtained through “leave-one-out” cross-validation (LOOCV). T25FWT, Timed25-Foot Walk Test- leg function; 9-HPT, 9-Hole Peg Test—arm function; PASAT, paced auditory serial addition test; SRT-LTS, selective reminding test-long-term storage; SRT-CLTR, selective reminding test-consistent long-term storage; SRT-D, selective reminding test-delayed recall; SPART 10/36, spatial recall test; WLG, word list generation; SDMT, symbol digit modality test. Bold values indicates the statistically significant values*.

Specifically, changes in 9-HPT scores were associated with the gender (*p* < 0.05), number (*p* < 0.05) and volume (*p* < 0.01) of new lesions, number of enlarged lesions (*p* < 0.05) and number of shrunken lesions (*p* < 0.05), Table 1 Supplementary data.

Changes in PASAT (sustained attention, auditory information, processing speed, and flexibility) score were significantly associated with the patients age (*p* < 0.01) and number of CL/WML that shrunk in size (*p* < 0.05), Table 2 Supplementary data.

Changes in SRT-D were mainly associated with resolved CL/WML volume (*p* < 0.001), stable CL/WML volume (*p* < 0.001), new CL/WML number (*p* < 0.05), resolved CL/WML number (*p* < 0.001), sex (*p* < 0.01), Table 3 Supplementary data.

Changes in WLG test (semantic verbal fluency) was associated to the shrunken CL/WML volume (*p* < 0.01), stable CL/WML volume (*p* < 0.001), shrunken CL/WML number (*p* < 0.05), stable CL/WML number (*p* < 0.01), Hospital Anxiety and Depression scale-Depression (*p* < 0.05), Table 4 Supplementary data.

## Discussion

Our work shows that the number and volume of focal CL and WML are moderately related to neuroaxonal damage—as measured by sNfL—at early MS stages. We also determined that the changes in CL/WML load are associated with changes in cognition and in motor performance in our cohort of patients with short disease duration and on stable therapy.

MS is characterized by multifocal inflammatory processes, which lead to the formation of demyelinating lesions in cortical gray and white matter. These inflammatory processes dominate in early stages of the disease and can be targeted by current anti-inflammatory treatments (39), thereby slowing the accumulation of disability (40). Hence, early biomarkers of ongoing disease activity are fundamental to judge on the need of therapy-switch and escalation at early disease stages (41).

In this work, we have studied patients with early RRMS and mild physical disability, who were on first-line treatment at time of enrollment.

We assessed whether CL and WML load and their changes over 2 years might be a useful biomarker to quantify neuroaxonal damage in those patients. To assess neurodegeneration, we used a serum biomarker i.e., sNfL, since a previous study in the same cohort showed the absence of brain atrophy over the 2-years follow-up (42).

We found a moderate correlation between CL and WML load at baseline and sNfL measures at the same time point, confirming and extending previous knowledge that focal WM lesions affect overall neuroaxonal damage in patients with MS (43, 44). The measure of sNfL levels at baseline also showed a correlation with the increase in WML number over 2 years. These findings confirm and extend previous knowledge that sNfL levels are related to WML volume at 2 years follow-up in MS patient at more advanced disease stage (43); additionally, these data suggest that sNfL measurements at baseline may provide important complementary information over WM disease activity during the 2 years that follow but not of CL activity.

Interestingly, we did not measure any significant correlation between sNfL and changes in CL at 2 years follow-up, which is probably due to the low number of CL compared to WML in our cohort of patients.

We also showed that changes in size and number of lesions were strongly associated with changes in cognition (sustained attention, processing speed and flexibility as well as in spatial memory and semantic verbal fluency) but also with changes in hand motor function. It is known that some lesions—especially the recent ones—may shrink in size over time and their intensity on T2-weighted (i.e., FLAIR) images decreases as edema resolves and some tissue repair occurs, leaving a smaller lesion or an undetectable plaque (45). Other lesions undergo little changes in size (stable lesions) and some others significantly increase in volume over-time (e.g., lesions with chronic activity) (46). Much is known about the relationship between new and enlarging lesions and clinical outcome in MS (47, 48) but there is currently little knowledge about the contribution of shrinking and resolving lesions. Our results provide a new window into the complex changes in CL and WML, which influence mild changes in cognition and motor function in early MS patients on therapy.

Remarkably, our data also provide evidence that the reparatory activity in focal plaques– as measured through the number and volume of resolved and shrunk lesions—appear to strongly correlate with cognitive changes in our cohort of patients. Since a comprehensive cognitive assessment in clinical practice may be time consuming and unrealistic for routine follow-up of MS patients, the detection of new CL and WML during the early stage of the disease may support with alternative monitoring tools.

Detection of CL and of changes in WML and CL load in clinical practice is challenging. We have assessed the number and volume of cortical lesions and their changes over time by using MP2RAGE; this is a clinically available MR sequence that has shown similar sensitivity to double inversion recovery (DIR) for CL detection (36) and that appears to be artifact free in contrast to DIR (49). MP2RAGE may therefore provide the opportunity—together with a 3D FLAIR sequence for optimal WML detection– to assess the overall burden of focal activity in early MS patients in clinical practice.

Limitations of this study are the relatively small and homogeneous sample size and the fact that, due to the moderate number of patients studied, we could not consider treatment as a covariate in our regression models. We also acknowledge that the absence of information about gadolinium-enhancement at the time of the MRI might have influenced the sNFL results although this is not highly probable since patients were clinically stable and on therapy. In addition, we did not have a matched population of healthy controls to determine whether the measured sNFL levels were increased in patients. Future work should confirm these finding in larger cohorts of patients, including subjects with higher disability scores and disease activity as well as healthy controls.

In summary, our results suggest that early assessment of CL/WML load and their short-term evolution during the first year of disease are sensitive to ongoing axonal damage and related to subtle clinical changes. New efforts should be devoted to using these metrics to stratify patients at the beginning of the disease and hence to identify the ones who need more aggressive first-line therapies or therapeutic escalation.

## Data Availability Statement

Images and detailed clinical scores may be available upon reasonable request.

## Ethics Statement

The studies involving human participants were reviewed and approved by Ethic committee of Lausanne University Hospital. The patients/participants provided their written informed consent to participate in this study.

## Author Contributions

CG: conceptualization, funding acquisition, and supervision. CG, P-JL, MF, R-AT, and GK: methodology. CG, P-JL, MF, and R-AT: formal analysis and investigation. R-AT, P-JL, and CG: writing—original draft preparation. R-AT, CG, MF, GB, RD, GK, MB, MP, LK, and JK: writing—review and editing. All authors contributed to the article and approved the submitted version.

## Conflict of Interest

GK and MF works for Siemens AG, Switzerland. The remaining authors declare that the research was conducted in the absence of any commercial or financial relationships that could be construed as a potential conflict of interest.
