# Supplementary material for: Evolution of Cortical and White Matter Lesion Load in Early-Stage Multiple Sclerosis: Correlation With Neuroaxonal Damage and Clinical Changes
Source: Front Neurol. 2020 Sep 4;11:973. doi: 10.3389/fneur.2020.00973 (PMC7498574; doi:10.3389/fneur.2020.00973)
Supplement: Supplementary file 1 [file Table_1.docx]

**Table 1.** Multiple regression between MRI metrics and 9-HPT (9-Hole Peg Test).

| **Coefficients** |  | | | |
| --- | --- | --- | --- | --- |
|  | Estimate | Std.Error | T value | P value |
| Sex | 1.672 | 0.622 | 2.687 | 0.05 |
| New_volume | -7.348 | 2.225 | -3.302 | 0.01 |
| New_count | 0.334 | 0.141 | 2.401 | 0.05 |
| Enlarged_count | -0.245 | 0.112 | -2.185 | 0.05 |
| Shrunken_count | -0.729 | 0.297 | -2.452 | 0.05 |
| 9-HPT: 9-Hole Peg Test (Motor hand function)  Residual standard error: 1.538 on 22 degrees of freedom  Multiple R-squared: 0.6277,  Adjusted R-squared: 0.4754  F-statistic: 4.121 on 9 and 22 DF, p-value: 0.003215 | | | | |

**Table 2.** Multiple regression between MRI metrics and PASAT (paced auditory serial addition test).

| **Coefficients** |  | | | |
| --- | --- | --- | --- | --- |
|  | Estimate | Std.Error | T value | P value |
| Age | -0.079 | 0.021 | -3.66 | **0.01** |
| No. resolved lesions | 0.071 | 0.037 | 1.888 | 0.1 |
| No. shrunk lesions | -0.518 | 0.214 | -2.421 | **0.05** |
| PASAT: paced auditory serial addition test  Residual standard error: 1.17 on 25 degrees of freedom. Multiple R-squared:  0.563    Adjusted R-squared:  0.458. F-statistic: 5.38 on 6 and 25 DF, p-value: 0.0011 | | | | |

**Table 3.** Multiple regression between MRI metrics and SRT-D (selective reminding test-delayed recall)

| **Coefficients** |  | | | |
| --- | --- | --- | --- | --- |
|  | Estimate | Std.Error | T value | P value |
| Sex | 0.715 | 0.229 | 3.123 | 0.01 |
| Resolved lesions_volume | 5.611 | 1.274 | 4.402 | 0.001 |
| Stable lesions_volume | 0.134 | 0.031 | 4.335 | 0.001 |
| New lesions_count | 0.128 | 0.054 | 2.334 | 0.05 |
| Resolved lesions_count | -0.189 | 0.0395 | -4.801 | -0.001 |
| SRT-D: selective reminding test-delayed recall  Residual standard error: 0.5892 on 23 degrees of freedom  Multiple R-squared: 0.678, Adjusted R-squared: 0.566  F-statistic: 6.054 on 8 and 23 DF, p-value: 0.0003122 | | | | |

**Table 4.**  Multiple regression between MRI metrics and WLG test (word list generation test)

| **Coefficients** |  | | | |
| --- | --- | --- | --- | --- |
|  | Estimate | Std.Error | T value | P value |
| Shrunken lesions_volume | -4.935 | 1.638 | -3.013 | 0.01 |
| Stable lesions_volume | -1.002 | 0.251 | -3.991 | 0.001 |
| Shruncken lesions_count | 2.587 | 0.955 | 2.708 | 0.05 |
| Stable lesions_count | 0.126 | 0.043 | 2.932 | 0.01 |
| HAD-D | -0.819 | 0.342 | -2.396 | 0.05 |
| WLG- word list generation test,  Residual standard error: 4.07 on 24 degrees of freedom  Multiple R-squared: 0.5596, Adjusted R-squared: 0.4312  F-statistic: 4.357 on 7 and 24 DF, p-value: 0.003052 | | | | |
